# Rectangular Improvement Method for Plan View Pattern of Plates During the Angular Rolling Process

**DOI:** 10.3390/ma17235964

**Published:** 2024-12-05

**Authors:** Chunyu He, Junyi Luo, Zhipeng Xu, Zhiqiang Wang, Zhong Zhao, Zhiqiang Wu, Zhijie Jiao

**Affiliations:** State Key Laboratory of Rolling and Automation, Northeastern University, Shenyang 110819, China; luojuny0617@163.com (J.L.); 18325576566@163.com (Z.X.); wangzhiq1229@163.com (Z.W.); zhaozhong@ral.neu.edu.cn (Z.Z.); wuzq@ral.neu.edu.cn (Z.W.)

**Keywords:** plate, angular rolling, rectangular, numerical simulation, experimental study

## Abstract

The effect of the angular rolling process on the plan view pattern of a plate was studied, and the rectangular influencing factors and improvement methods for this process were proposed in this paper. DEFORM (v11.0) finite element software was used to simulate the processes of conventional rolling and angular rolling, and the degree of rectangularity of plates under different rolling process conditions was compared. A formula to characterize the degree of rectangularity of plates was established; the closer this value is to one, the better the degree of rectangularity. Considering the actual rolling process conditions, the range of theoretically calculated rectangular rotation angles was extended to obtain the optimum rectangular rotation angle using the finite element simulation method. In the two-pass angular rolling process, the optimal rectangular angle of the second pass was 14.275° when the first pass was 15°. The optimal rectangular angle of the plate was 19.008° when the first pass’ angle was 20°. Two-pass angular rolling is different to four-pass rolling, and the simulation results showed that $J_{\;15{}^{\circ}}^{\;4}$ (1.0012) was less than $J_{\;15{}^{\circ}}^{\;2}$ (1.0015) and $J_{\;20{}^{\circ}}^{\;4}$ (1.0034) was less than $J_{\;20{}^{\circ}}^{\;2}$ (1.0055). The rectangularity degree of the four-pass process was better than the two-pass process. Angular rolling experiments were carried out, and the actual data show that the characteristic rectangular value of the rolled piece was 1.003 during the four-pass process and 1.014 during the two-pass process. This verified that separating the one-group two-pass angular rolling process from the one-group four-pass angular rolling process can improve the rectangular degree of the rolled plate, thereby increasing the yield rate. This provides a theoretical basis for industrial applications.

## 1. Introduction

**Figure 1:**
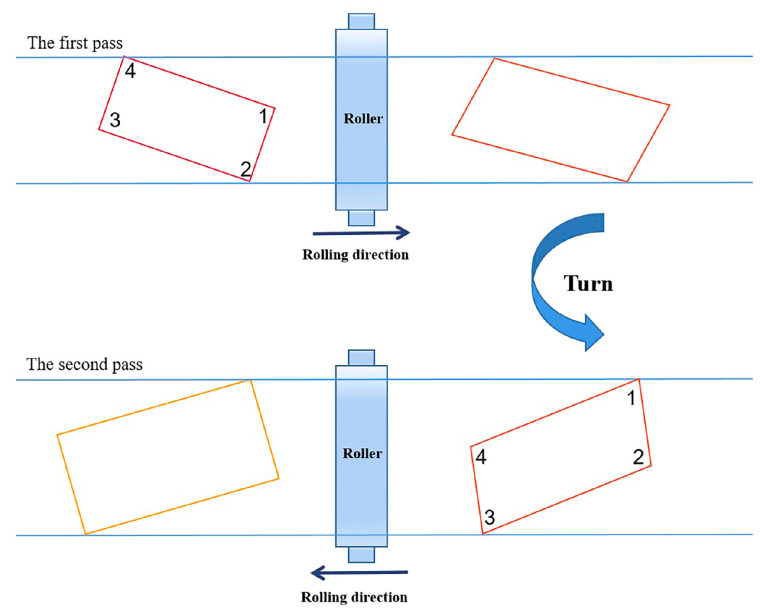
Schematic diagram of two-pass angular rolling.

The global steel industry has entered a new stage [1]. Medium- and heavy-plate steel, as important types of steel, have irreplaceable roles in national construction economics, national security, and other aspects [2]. With the continuous changes in customers’ demands, larger weighted plate units are needed [3]. When the conventional rolling process is used to produce large units of weighted products, the required length of a slab is larger than the equipment’s limit, so the current rolling mill capacity cannot meet the production requirements. In addition, in the production of plates with small spread ratios, the plate needs to be turned twice, as not doing so would reduce the production efficiency. These problems can be solved with the angular rolling process [4]. During the angular rolling production process, the center line of the slab is rolled away from the initial rolling direction at a specific angle [5,6]. Angular rolling is a rolling technique between broadening rolling and elongation rolling, as shown in Figure 1. During the angular rolling process, the rolled piece is first rotated to a certain angle to initially locate the No. 1 corner within the roll gap. The plan view pattern of the rolled piece is rolled to become a parallelogram. Then, the rolled piece is turned in order to locate the No. 4 corner within the roll gap. The plan view pattern of the rolled piece is rolled from a parallelogram to a rectangle again [7].

The angular rolling process was first studied by scientists in the former Soviet Union, where the width formula for angular rolling was proposed. In China, in the 1990s, Li proposed a new formula for angular rolling [8]. Cao et al. developed a calculation model and an automatic control system for the angular rolling process [9]. Cao et al. explored the method of rotation angle control and obtained empirical widening parameters [10]. During the development and theoretical study of the angular rolling process, Zhou studied the effects of angular rolling process on plate mechanical properties and the improvement in mill rolling conditions [4]. Hao et al. established a mathematical model based on the influence function method to calculate the thickness distribution and rolling force during angular rolling [11].

As demonstrated in the current research on plate rolling processes, using finite element software for simulation and analysis can greatly reduce research costs. DEFORM finite element software is specifically designed for metal forming and is a heat treatment process simulation software, covering all processes from metal solidification to heat treatment, microstructure changes, and so on, with a powerful three-dimensional deformation analysis ability. DEFORM uses a high-precision hexahedral mesh and advanced algorithm, which not only ensures the accuracy of the simulation but also shortens the calculation time. Data similar to real-world situations can be obtained by establishing a reasonable finite element model. Finite element simulation studies have achieved good results regarding the evolution behavior of voids [12], the prediction of the plan view pattern, the shape changing rule during plate rolling [13,14,15,16], and the width spreading of angular rolling [17,18]. DEFORM-3D, a finite element software which is suitable for rolling process studies, has also been applied to different aspects of strip and plate rolling process studies. Simultaneously, the experimental validation results demonstrated the reliability of numerical simulations using DEFORM [19,20,21,22,23].

The main problem that occurs during the practical application of angular rolling technology is that the rectangular pattern is difficult to control. The rectangularity degree is closely related to the yield rate, which is an important factor affecting economic indicators. The method used to improve the rectangularity degree in conventional rolling mainly involves plan view pattern control technology [24]. Jiao et al. developed a mathematical model for plan view pattern prediction and online control [25,26,27], while Shigemori et al. proposed a parameter identification technique with a locally weighted regression model for the plan view pattern control of the plates [28].

The plan view pattern control method used in conventional rolling technology is difficult to apply to the angular rolling process. Finding ways through which to improve the plan view pattern of the plate after angular rolling is a tough problem in current research. Hao proposed a method to predict the plan view pattern of the plate after angular rolling by segmenting the plate [29]. Jiao et al. used the trigonometric function and the principle of constant volume to predict the plan view pattern changing during the two-pass angular rolling process and investigated the relationship between the two passes that still reverts to a rectangular pattern after a two-pass process, under ideal conditions [30,31].

However, the two-pass angular rolling processes described above are based on ideal conditions, where the biting and throwing stages are considered symmetrical. The difference between the biting and throwing stages of the plate deformation is ignored. In the actual production process, it is difficult to ensure that the plane pattern of the rolled piece is completely symmetrical after it undergoes the two-pass angular rolling process.

**Figure 2:**
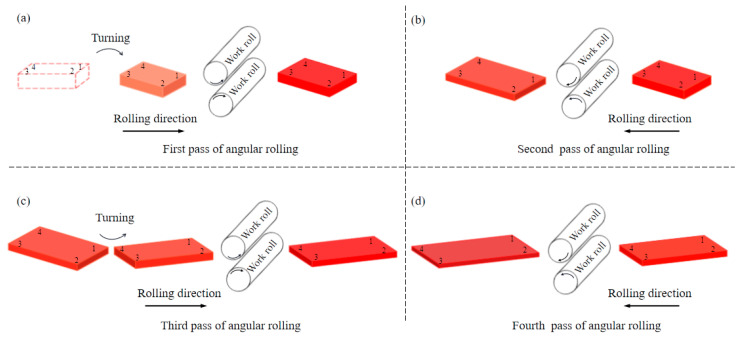
Schematic diagram of four-pass angular rolling: (**a**) first pass; (**b**) second pass; (**c**) third pass; (**d**) fourth pass.

When the two-pass angular rolling process is adopted, because the first bite is into the two corners on the same side as the lengthwise direction, different metal flow degrees on both sides lead to uneven deformation, which affects the rectangularity effect of the plate. In this paper, based on the difference between the biting and throwing stages of plate deformation, the two-pass angular rolling process is transformed into a four-pass angular rolling process. The whole process is shown in Figure 2. Corner 1 is first bitten into the roll gap, then a reverse rolling pass is carried out without turning the rolled piece. Corner 3 is rolled, and the rolled piece becomes a parallelogram. Then, the rolled piece is turned, and corner 2 is rolled. Finally, another reverse rolling pass is carried out, corner 4 is rolled, and the rolled piece becomes a rectangle again. In this process, each corner of the rolled piece was rolled through a biting phase and a throwing phase. With this process, the plan view pattern after angular rolling may be improved.

In this paper, the effect of the angular rolling process on the plan view pattern of the plate was studied. In the second part of this paper, conventional rolling and angular rolling processes were investigated using the finite element numerical simulation method. The formula for characteristic rectangular value was proposed, in order to characterize the rectangularity degree of the rolled plate. In the third part of this paper, the effects of the one-group two- and four-pass angular rolling processes on the plan view pattern of rolled pieces were analyzed. In the fourth part, the simulation results were further validated through rolling experiments.

## 2. Numerical Simulation Research on Angular Rolling Processes

The finite element software DEFORM-3D is used for numerical simulation research into of the rolling process. In the pre-processing module, the geometric model is created, meshing is performed, and the main parameters are set.

### 2.1. Numerical Simulation Modeling and Parameter Setting

#### 2.1.1. Geometric Model

**Figure 3:**
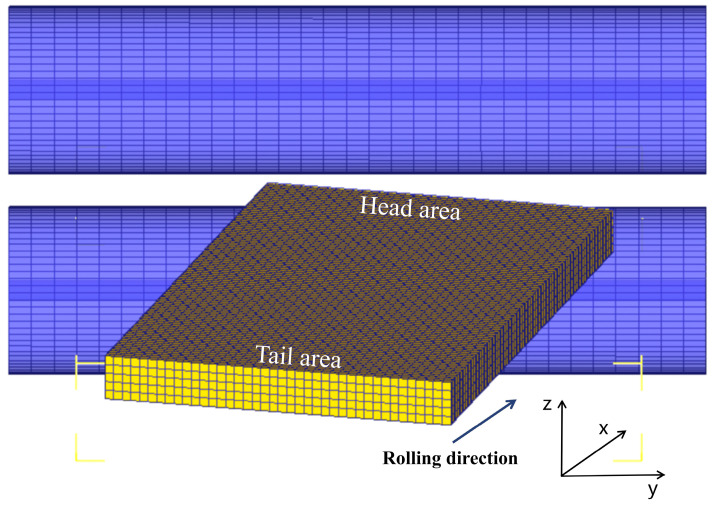
Schematic diagram of the geometric model.

Angular rolling is asymmetric rolling, so the whole plate and two work rolls should be modeled in the same way as a geometric model. The work rolls, plate, and pusher were all modeled using the DEFORM-3D modeling module. The geometric model after creation is shown in Figure 3.

#### 2.1.2. Simulation Conditions

After establishing a 3D model of the angular rolling process in the pre-processing DEFORM module, we set the relevant parameters for angular rolling. After the simulation was initiated, the software ran FEM code to analyze the rolling process. The simulation results can be viewed in the post-processing module of DEFORM. The input parameters used for the DEFORM-3D simulation are given in Table 1.

### 2.2. Numerical Simulation Scheme

#### 2.2.1. Conventional Rolling Simulation Scheme

In order to study the plate deformation difference between the biting and throwing stages under conventional conditions, a set of conventional rolling simulations were set up to simulate the deformation of the head and tail during rolling.

The simulation slab size was 320 × 2570 × 4500 mm, which was similar to the slab size used in actual production, while the pass reduction rate was set to 30%.

#### 2.2.2. Angular Rolling Simulation Scheme

Using the angular rolling plan pattern prediction model presented in the literature [32], the rotation angle of the second pass required for two-pass angular rolling can be calculated. However, the model describes ideal angular rolling deformation behavior, meaning that the difference in metal flow between the head and tail parts of the rolled piece is ignored. Therefore, actual rolling conditions should be considered when using these formulas to calculate the simulation pass schedule data.

The simulation slab size specifications were as follows: thickness: 320 mm; width: 2570 mm; and length: 4500 mm. The total reduction was set as 40 mm. One-group two-pass angular rolling and one-group four-pass angular rolling simulations with the same pass reduction ratio were simulated. The pass reduction ratio was 6.46% and 3.28% for two-pass angular rolling and four-pass angular rolling, respectively.

Combined with the plant equipment size and production efficiency, when the current angular rolling rotation angle is usually between 15° and 20°, it can not only increase the width of large single-weight long-size billets but also reduce the time required for complete steel transformation and improve production efficiency. The first rotation angles for the two angular rolling processes are set at 15° and 20°, respectively. According to Formula (2), it can be calculated that the second pass angles, satisfying the condition of rectangularization, are 14.075° and 18.808°, respectively.

(1) $$\frac{b_{2}^{2}}{b_{0}^{2}}=\left(\frac{h_{0}^{2}}{h_{1}^{2}}\sin^{2}\alpha +\cos^{2}\alpha \right)\left(\frac{h_{1}^{2}}{h_{2}^{2}}\sin^{2}\beta +\cos^{2}\beta \right)$$

The rotation angle of the first angular rolling pass is *α*. The rotation angle of the second pass is *β*, *b*_0_ is the slab width, *b*_2_ is the width after the second pass, *h*_0_ is the initial slab thickness, and *h*_1_ is the plate thickness after the first pass. Table 2 shows the second pass rotation angle calculated with theoretical model.

The values calculated by the theoretical model will deviate from the actual values. To be closer to the actual rolling conditions, the rotation angle of the second pass was expanded for simulations based on the calculation result. Table 3 shows the simulation rotation angle for the first case. Also, the second case of a 20° first pass rotation angle was expanded using the same schema.

Because there are many factors affecting the plan view pattern of diagonal rolling in industrial production, the numerical value calculated by the theoretical model will be different from the actual value. In order to more closely model the actual rolling situation, the rotation angle range of the second pass was extended on the basis of the theoretical calculation results, and 42 sets of (±1°) simulations were carried out at intervals of 0.1°. Table 3 shows the rotation angle used in the second-run simulation, when the first pass was set to 15°. At the same time, a simulation at the same interval was carried out for the first rotation angle of 20°, and the theoretical calculation value of the second pass was 18.808°.

The best angle of rotation for rectangles was found using simulations for two cases. One-group four-pass angular rolling was simulated using this angle to compare the rectangularity degree.

### 2.3. Characteristic Rectangular Values

To facilitate the comparison of rectangularity, a parameter characterizing the rectangularity degree needs to be defined.

**Figure 4:**
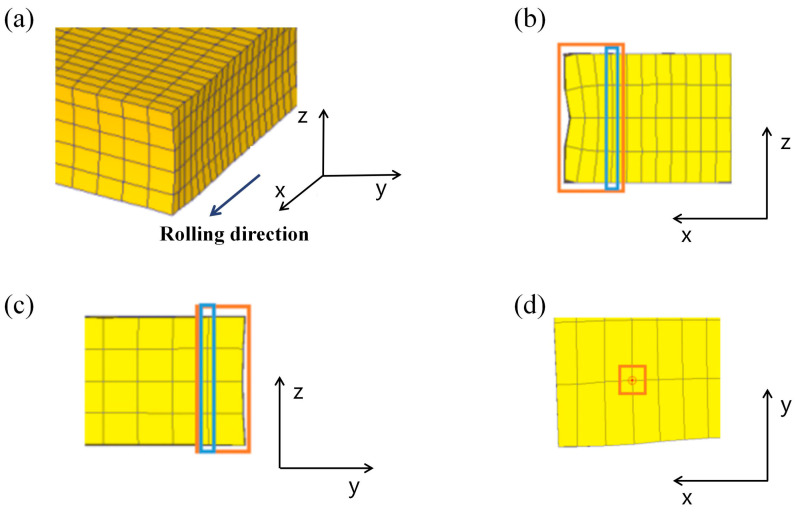
Lower right corner after rolling: (**a**) lower right corner three-dimensional (3D) view; (**b**) lower right corner x–z view; (**c**) lower right corner y–z view; (**d**) lower right corner x–y view.

After the slab was rolled, the edges were deformed to a certain extent, as shown in Figure 4. It can be seen from Figure 4a that the edges had a crocodile mouth defect, which may lead to errors in the results if the points on the edges are directly selected for analysis. To avoid this problem, the points in the edge distortion area should not be selected. As can be seen in Figure 4b, the distortion was already more moderate on the right side of the third column of the mesh from left to right and, in Figure 4c, on the left side of the first column of the mesh from right to left. Extending the first line along the y-axis into a face and the second line along the x-axis into a face, the point where the two faces intersect was taken as one of the selected points, as shown in Figure 4d. The other three points were selected using the same principle in the other three corners.

**Figure 5:**
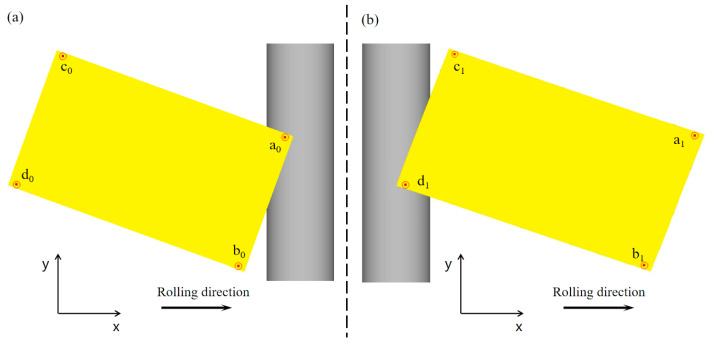
Pointing diagram for angular rolled parameters: (**a**) before the first pass and (**b**) after the first pass.

After the points were selected, they were marked as shown in Figure 5. Subscripts 0, 1, and 2 indicate the raw material stage, the first pass, and the second pass of the rolling stage, respectively. For example, a_0_b_0_ indicates the length of the ab side of the raw material, and a1c1 indicates the length of the ac side after the first pass is completed.

Considering that the corner angles of the rolled piece are more difficult to measure after angular rolling, a characteristic rectangular value representing the rectangularity degree was defined using the length, width, and diagonal length and can be expressed as in Equation (1) as follows:

(2) $$J_{\alpha }^{n}=\frac{\mathrm{cd}}{\mathrm{ab}}\times \frac{\mathrm{bd}}{\mathrm{ac}}\times \frac{\mathrm{ad}}{\mathrm{bc}}$$

In the above equation, *J* represents the characteristic rectangular value. *n* is the total number of angular rolling passes. The rotation angle of the first angular rolling pass is *α*. The closer this value is to 1 (*J* equals 1 for rectangles), the better the rectangularity degree.

## 3. Simulation Result Analysis

### 3.1. Conventional Rolling Simulation Results

**Figure 6:**
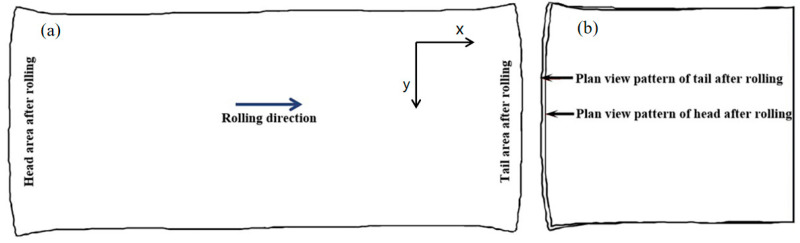
Conventional rolling deformation diagram: (**a**) plan view pattern after rolling and (**b**) comparison of the plan view pattern of the head and tail along the center line after rolling.

**Figure 7:**
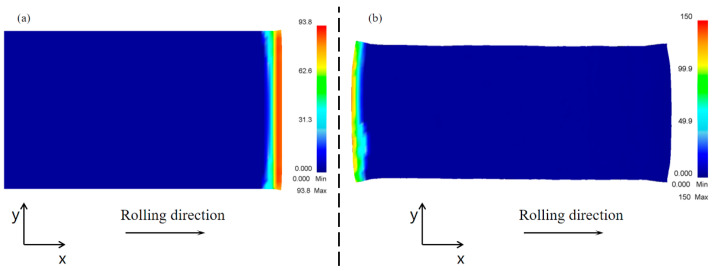
Stress diagram for conventional rolling biting and throwing stages: (**a**) rolling biting stage and (**b**) rolling throwing stage.

The plan view pattern after the conventional rolling process is shown in Figure 6. Comparing the plan view patterns of the head and tail of the rolled piece, the head deformation of the rolled piece is smaller than its tail deformation, which indicates that there was a difference between the deformation behavior of the rolled piece in the biting stage and the throwing stage. Figure 7 shows that the rolling stresses in the biting stage are less than those in the throwing stage; these higher stresses can produce more deformation. In addition, according to the analysis of the metal flow during the rolling process, when the head of the rolled piece is bitten, the metal flow is backward along the rolling direction, towards the back of the overall rolled piece. When the tail of the rolled piece is thrown, the metal is still experiencing backward flow, at the back of the free end. Compared with the head, the tail is more conducive to metal flow and can, therefore, induce more deformation.

### 3.2. Angular Rolling Simulation Results

**Figure 8:**
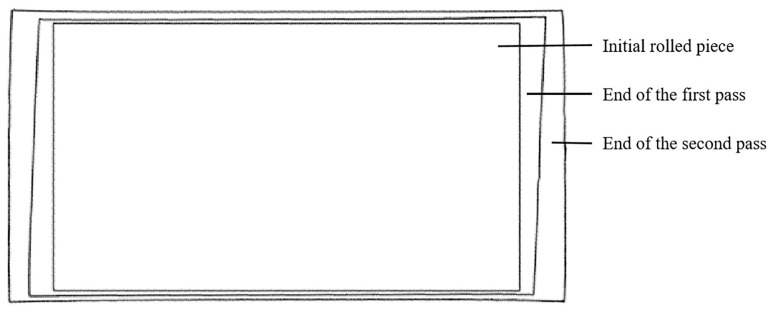
Evolution of the plan view pattern of the rolled piece by two-pass angular rolling.

**Figure 9:**
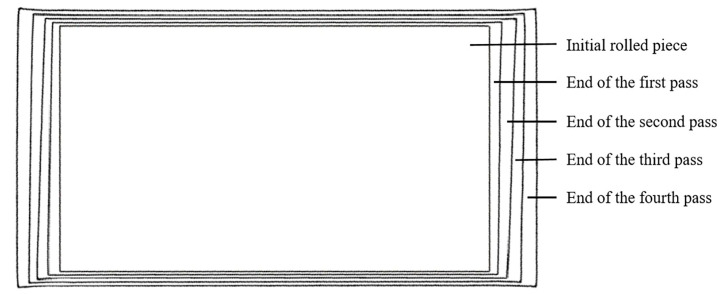
Evolution of the plan view pattern of the rolled piece by four-pass angular rolling.

The plan view patterns simulated after each pass of the two different processes were obtained and compared; these comparisons are shown in Figure 8 and Figure 9. The innermost pattern in the figure is the initial plan view pattern of the rolled piece, the pattern in the middle is the plan view pattern after each pass of angular rolling, and the outermost one is the final plan view pattern after rolling.

After the simulation, all the data were recorded, according to the above selected points.

### 3.3. Rectangularity Degree Comparison

**Figure 10:**
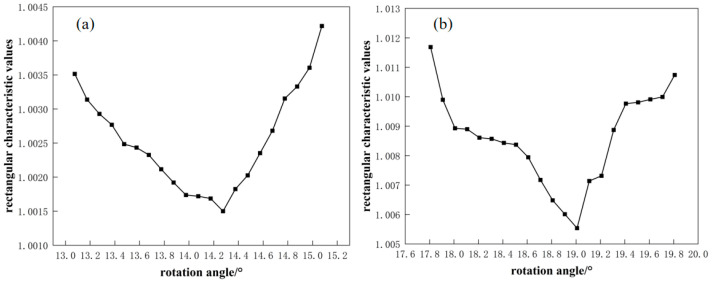
Characteristic rectangular values of two-pass angular rolling: (**a**) first pass rotated by 15° and (**b**) first pass rotated by 20°.

After substituting the simulation results into Equation (1), the results are obtained as shown in Figure 10.

The simulated results of 42 lengths in the two groups of different angular rolling processes were substituted into Equation (1), and the calculated values were plotted as shown in Figure 10. When the angle gradually increased and approached the theoretical value, the characteristic rectangular value gradually decreased, the plate changed from parallelogram to rectangle, and the rectangularity effect was improved. The optimal rotation angle of rectangularization is slightly larger than the theoretical value. When the rotation angle continued to increase, the characteristic rectangular value gradually increased, the plate gradually changed from a rectangle to a parallelogram, and the rectangularity effect decreased.

The best rotation angles of the second pass and best characteristic rectangular values corresponding to the rotation angles of the first pass are shown in Table 4. When the first rotation angle of angular rolling was 15°, the theoretical value was 14.075°, the optimal rotation angle obtained by the simulation was 14.275°, the difference was 0.2°, and the error was 1.4%. ${J\;}_{15{}^{\circ}}^{\;2}$ was 1.0015. When the first rotation angle of angular rolling was 20°, the theoretical calculation value was 18.808°, and the optimal rotation angle obtained by simulation was 19.008°, the difference was 0.2°, and the error was 1.1%. ${J\;}_{20{}^{\circ}}^{\;2}$ was 1.0055.

The optimal angles obtained by the two groups of simulation are greater than the theoretical calculation value, indicating that the rotation angles calculated by the theoretical calculation have some errors in the actual rolling process; however, the error value is small, the theoretical accuracy is high, and it has practical application value.

The purpose of the above simulation was to obtain the rotation angle with the best resulting rectangularity effect in the two-pass angular rolling process. This angle was used to simulate the four-pass angular rolling process and compare the rectangularity effect. The rotation angles of each of the four-pass angular rolling after the decomposition of the two groups are shown in Table 5.

The best rotation angle for the two-pass angular rolling process was applied to the simulation of the four-pass angular rolling process, and the simulation values were substituted into Equation (1), resulting in the following:

$$J_{15{}^{\circ}}^{4}=1.0012$$

$$J_{20{}^{\circ}}^{4}=1.0034$$

We compared the best characteristic rectangular values of the two-pass angular rolling process with the corresponding characteristic rectangular values of the four-pass angular rolling process, as shown in Table 6. $J_{\;15{}^{\circ}}^{\;4}$ was less than $J_{\;15{}^{\circ}}^{\;2}$ and $J_{\;20{}^{\circ}}^{\;4}$ was less than $J_{\;20{}^{\circ}}^{\;2}$. It was found that the four-pass angular rolling process is better than the two-pass angular rolling process, regarding the rectangularity degree.

## 4. Validation Experiment

### 4.1. Experimental Procedure

**Figure 11:**
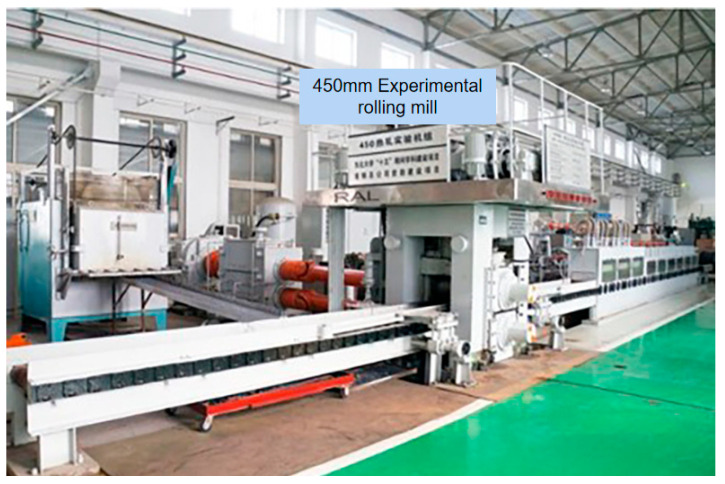
Experimental rolling mill.

An experiment comparing the rectangularity degrees resulting from the use of the two- and four-pass angular rolling processes was carried out on a 450 mm hot rolling experimental mill at the State Key Laboratory of Rolling and Automation at Northeastern University. The experimental equipment is shown in Figure 11. The experimental material was two pure lead blocks, which demonstrate deformation behavior at room temperature that was close to that of the high-temperature plate. A rectangle was drawn 2 mm inside the edge of the specimen for measurement after rolling. The rolling mill equipment parameters and rolled piece size data are shown in Table 7.

When the rotation angle of the first pass of angular rolling was 20°, the angle of the second pass of angular rolling required to meet the rectangular condition was calculated to be 17.5°, based on the size of the experimental billet. The experimental rolling schedules are shown in Table 8.

### 4.2. Experimental Result

**Figure 12:**
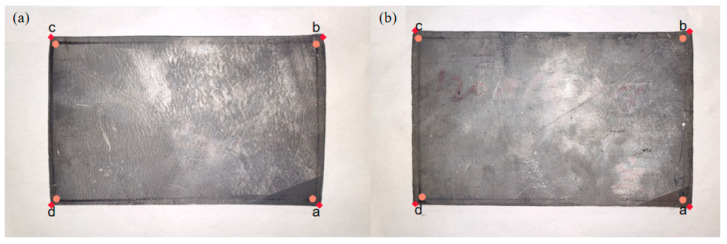
Plan view pattern after angular rolling (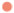
–reference points, 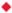
–edge points): (**a**) two-pass angular rolling and (**b**) four-pass angular rolling.

The plan view patterns after the two- and four-pass angular rolling processes are shown in Figure 12, where point a is the first bitten corner during angular rolling. Table 9 shows the dimensional data of the two processes after rolling (the edge points are the four vertices of the rolled piece surface, and the reference points are the four vertices of the rectangle connected by parallel lines 2 mm inwards from each side of the rolled piece surface).

**Figure 13:**
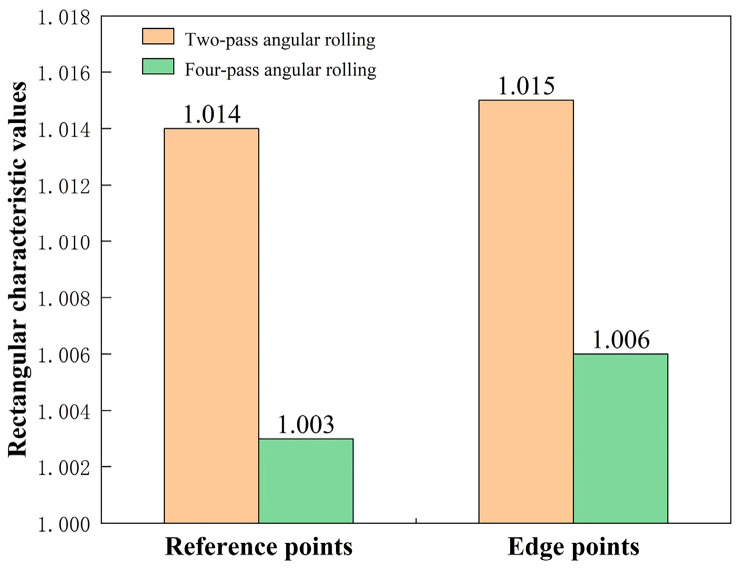
Comparison of the characteristic rectangular values of the two processes.

The experimental data were substituted into Formula (2), and the calculation results are shown in Figure 13. The characteristic rectangular values obtained using reference points and edge points of the two-pass angular rolling plate were 1.014 and 1.015, respectively. The characteristic rectangular values obtained using reference points and edge points of the four-pass angular rolling plate were 1.003 and 1.006, respectively. According to the explanation of Formula (2), the characteristic rectangular value resulting from four-pass angular rolling was closer to one. The experimental data show that the rectangularity effect of four-pass angular rolling was better than that of two-pass angular rolling. Therefore, the experimental results were consistent with the finite element simulation results.

Through the detailed analysis of the comprehensive simulation and experimental results, we found that, when implementing two-pass angular rolling, the parts of the plate that first entered the mill were the two corners on the same lengthwise side, which led to a significant increase in the metal flow of the plate on that side. In contrast, the flow was weaker on the other side. The plate showed uneven deformation on both sides in the lengthwise direction. When four-pass angular rolling was implemented, all four corners of the plate could be rolled first, which effectively alleviated the problem of uneven deformation in two-pass angular rolling and significantly increased the yield.

In view of the physical size limitation of the experimental rolling mill equipment, in order to simulate actual production conditions to the greatest extent, the size of the billet used in the experiment was rationally reduced in proportion to the size of the billet that would be used in industrial production. At the same time, the key parameters involved in the experiment, such as the rotation angle and reduction rate, were strictly selected and set according to the actual industrial production conditions. In this study, the advantages of four-pass angular rolling regarding improving the rectangularity effect of the plate were verified successfully by the experimental rolling mill. This study has thus laid a solid theoretical and experimental foundation for the practical application of this process in industrial production.

## 5. Conclusions

The reason for the difference between the head and tail deformation of rolled pieces after conventional rolling was analyzed through numerical simulation. Since the two bites of the two-pass angular rolling process started from two corners on the same side, the deformation difference in the rolled piece between the biting and throwing stage affected the rectangularity degree of the two-pass angular rolled plate. It was proposed to modify the two-pass angular rolling process to a four-pass angular rolling process to improve the rectangle of the angular rolled plate.

A formula to calculate the characteristic rectangular values reflecting the rectangular degree of the plate was constructed. Through this numerical simulation, the deformation of the rolled piece under the optimal rectangular process conditions of two- and four-pass angular rolling was analyzed and compared. The simulation results showed that the rectangularity degree obtained using four-pass angular rolling was better than that obtained using two-pass angular rolling.

The angular rolling processes were experimentally verified. The characteristic rectangular value of the reference point of the four-pass angular rolled piece was 1.003, while that of the two-pass angular rolled piece was 1.014. The rectangle of the four-pass angular rolled piece was evidently better than that of the two-pass angular rolled piece.

The use of four-pass angular rolling can improve the degree of plate rectangularity, reduce the cutting loss, and increase the yield without increasing the turning time, as well as increase the profits of enterprises in the face of fierce competition in the plate market.

## Figures and Tables

**Table 1:** Simulation parameter settings.

DEFORM 3D Input Parameter	Typical Value
Units	SI
Solution type	Deformation + heat transfer
Rolling type	Lagrangian (incremental)
Coefficient of heat transfer	5
Roll material type	Rigid
Roll diameter	1200 mm
Roll barrel length	5000 mm
Initial length of slab	4500 mm
Slab temperature	1100 °C
Initial width of slab	2570 mm
Initial thickness of slab	320 mm
Number of meshing elements	24,846
Coefficient of friction	0.4
Roll temperature	40 °C
Rolling speed	25 rad/min

**Table 2:** Rotation angle pass schedule of two-pass angular rolling.

Group Number	1	2
First pass rotation angle/°	15	20
Second pass rotation angle/°	14.075	18.808

**Table 3:** Simulation rotation angle values.

No.	First Pass Rotation Angle/°	Second Pass Rotation Angle/°
1	15	13.075
2		13.175
3		13.275
4		13.375
5		13.475
6		13.575
7		13.675
8		13.775
9		13.875
10		13.975
11		14.075
12		14.175
13		14.275
14		14.375
15		14.475
16		14.575
17		14.675
18		14.775
19		14.875
20		14.975
21		15.075

**Table 4:** Characteristic rectangular values obtained from the simulation.

Rotation Angle of the First Pass/°	Optimum Rotation Angle of the Second Pass/°	Characteristic Rectangular Value
15	14.275	1.0015
20	19.008	1.0055

**Table 5:** Simulation rotation angle values.

Group Number	1	2
First pass rotation angle/°	15	20
Second pass rotation angle/°	15	20
Third pass rotation angle/°	14.275	19.008
Fourth pass rotation angle/°	14.275	19.008

**Table 6:** Simulation rotation angle values.

Process	Rotation Angle of the First Pass/°	*J*
Two-pass angular rolling	15	1.0015
Four-pass angular rolling	15	1.0012
Two-pass angular rolling	20	1.0055
Four-pass angular rolling	20	1.0034

**Table 7:** Rolling mill and mill parameters.

Parameter	Size
The diameter of roller/mm	450
The length of roller/mm	450
The length of rolled piece/mm	160
The width of rolled piece/mm	120
The thickness of rolled piece/mm	16

**Table 8:** Experimental rolling schedule.

Rolling Pass	Two-Pass	Four-Pass
Exit thickness of the first pass/mm	13.9	14.9
Exit thickness of the second pass/mm	12	13.9
Exit thickness of the third pass/mm	/	12.9
Exit thickness of the fourth pass/mm	/	12
Rotation angle of the first pass/°	20	20
Rotation angle of the second pass/°	17.5	20
Rotation angle of the third pass/°	/	17.5
Rotation angle of the fourth pass/°	/	17.5

**Table 9:** Experimental data.

Parameter	Two-Pass Angular Rolling (Edge Points)	Four-Pass Angular Rolling (Edge Points)	Two-Pass Angular Rolling (Reference Points)	Four-Pass Angular Rolling (Reference Points)
ab/mm	124.70	127.86	115.89	116.84
cd/mm	126.01	127.87	116.40	116.86
ad/mm	199.53	201.75	193.30	191.74
bc/mm	200.05	202.31	194.18	192.01
ac/mm	235.8	237.55	225.04	224.42
bd/mm	236.19	238.36	226.24	224.74

## Data Availability

The original contributions presented in this study are included in the article. Further inquiries can be directed to the corresponding author.
